# Direct and indirect associations between dietary magnesium intake and breast cancer risk

**DOI:** 10.1038/s41598-019-42282-y

**Published:** 2019-04-08

**Authors:** Wu-Qing Huang, Wei-Qing Long, Xiong-Fei Mo, Nai-Qi Zhang, Hong Luo, Fang-Yu Lin, Jing Huang, Cai-Xia Zhang

**Affiliations:** 1 Department of Medical Statistics and Epidemiology, School of Public Health, Sun Yat-sen University, Guangzhou, 510080 China; 2 Guangdong Provincial Key Laboratory of Food, Nutrition and Health, School of Public Health, Sun Yat-sen University, Guangzhou, 510080 China; 3 Department of Clinical Laboratory, the First Affiliated Hospital of Sun Yat-sen University, Guangzhou, 510080 China; 4 Department of Vascular Surgery, the First Affiliated Hospital of Sun Yat-sen University, Guangzhou, 510080 China; 5 Nursing Department, the First Affiliated Hospital of Sun Yat-sen University, Guangzhou, 510080 China

## Abstract

This study aimed to explore the effect of dietary magnesium intake on breast cancer risk both directly and indirectly via its effect on inflammatory markers C-reactive protein (CRP) and interleukin-6 (IL-6). This case-control study recruited 1050 case patients and 1229 control subjects. Inflammatory marker levels of 322 cases and 322 controls, randomly selected, were measured using ELISA, and data on dietary magnesium intake were collected using a food frequency questionnaire. Multivariable logistic regression was used to estimate the odds ratio (OR) and 95% confidence interval (CI), and path analysis was used to investigate the mediating effect. A higher magnesium intake was associated with a lower breast cancer risk (adjusted OR = 0.80, 95% CI = 0.65, 0.99). A positive association was found between the CRP level and breast cancer risk (adjusted OR = 1.43, 95% CI = 1.02–2.01). However, IL-6 was not found to be associated with breast cancer risk. Path analysis revealed that dietary magnesium affected breast cancer risk both directly and indirectly by influencing the CRP level. The results indicate that a direct negative association and an indirect association through influencing the CRP level were observed between dietary magnesium intake and breast cancer risk.

## Introduction

Breast cancer is the most common cancer among women, accounting for 25% of all female cancer cases^1^. Magnesium deficiency has been reported to be associated with the risk of some diseases, including cardiovascular disease, diabetes mellitus, metabolic syndrome, as well as various types of cancers^2–8^. Some studies have linked magnesium deficiency to the development and prognosis of breast cancer^2,9,10^, but this finding is inconsistent across studies. The bioavailability of magnesium depends largely on food sources^8^. Although a variety of foods and food groups, including green vegetables, beans and unrefined whole grains, are rich in magnesium, the daily intake of magnesium remains below the recommended daily allowance and does not meet even the estimated average requirement (EAR)^11^. The National Health and Nutrition Examination Survey reported that the magnesium intake of approximately 70% of American adults is insufficient because of increased consumption of refined foods, which are poor sources of magnesium^10,12^. Therefore, it is essential to investigate the effect of magnesium deficiency on breast cancer risk.

Magnesium plays essential roles in several biological reactions, such as inflammation, DNA replication and repair, cell proliferation and signalling transduction, most of which are linked to tumourigenesis^13^. In particular, there is evidence that magnesium deficiency is associated with inflammatory response, although the underlying mechanisms are still unclear^14–18^.

A possible relationship between inflammation and cancer was first suggested when Rudolf Virchow found the presence of leukocytes in tumours in 1863^19^. Although studies have suggested that the development of approximately 20% of all cancers is linked to chronic low-grade inflammation, the varying effects of inflammation on the onset of different types of cancer are still not completely clear^20,21^. Elevated levels of C-reactive protein (CRP) and several cytokines are associated with chronic low-grade inflammation^22,23^. The CRP level is widely used as a classic biomarker of systemic inflammation in epidemiological studies as it is sensitive to acute inflammation and also shows a moderate increase in chronic inflammation^24–26^. The use of CRP level as a chronic inflammatory biomarker has some advantages, such as wide availability of assays and temporal stability^27,28^. There is growing evidence that an increased CRP level is associated with the risk of colorectal and lung cancers, but evidence about the association of CRP level with breast cancer risk is inconsistent^29–35^. Interleukin-6 (IL-6) is a major pleiotropic pro-inflammatory cytokine that also reflects the systematic chronic inflammatory status. IL-6 bridges the signal transducer and activator of transcription 3 (Stat-3) and nuclear factor-kappa B (NF-κB)- dependent signalling pathways, which regulate both inflammatory response and tissue metabolism^36^. To date, very few epidemiological studies have assessed the role of IL-6 in the risk of breast cancer^37–39^.

Based on the above-mentioned findings, we speculated that magnesium deficiency affects breast cancer risk by regulating the systematic inflammatory status. Accordingly, this study aimed to investigate the direct association of dietary magnesium intake and inflammatory marker levels with breast cancer risk among Chinese women and to explore the indirect association of dietary magnesium with breast cancer risk through the modulation of chronic low-grade inflammation (i.e. changes in serum CRP and IL-6 levels).

## Results

**Figure 1:**
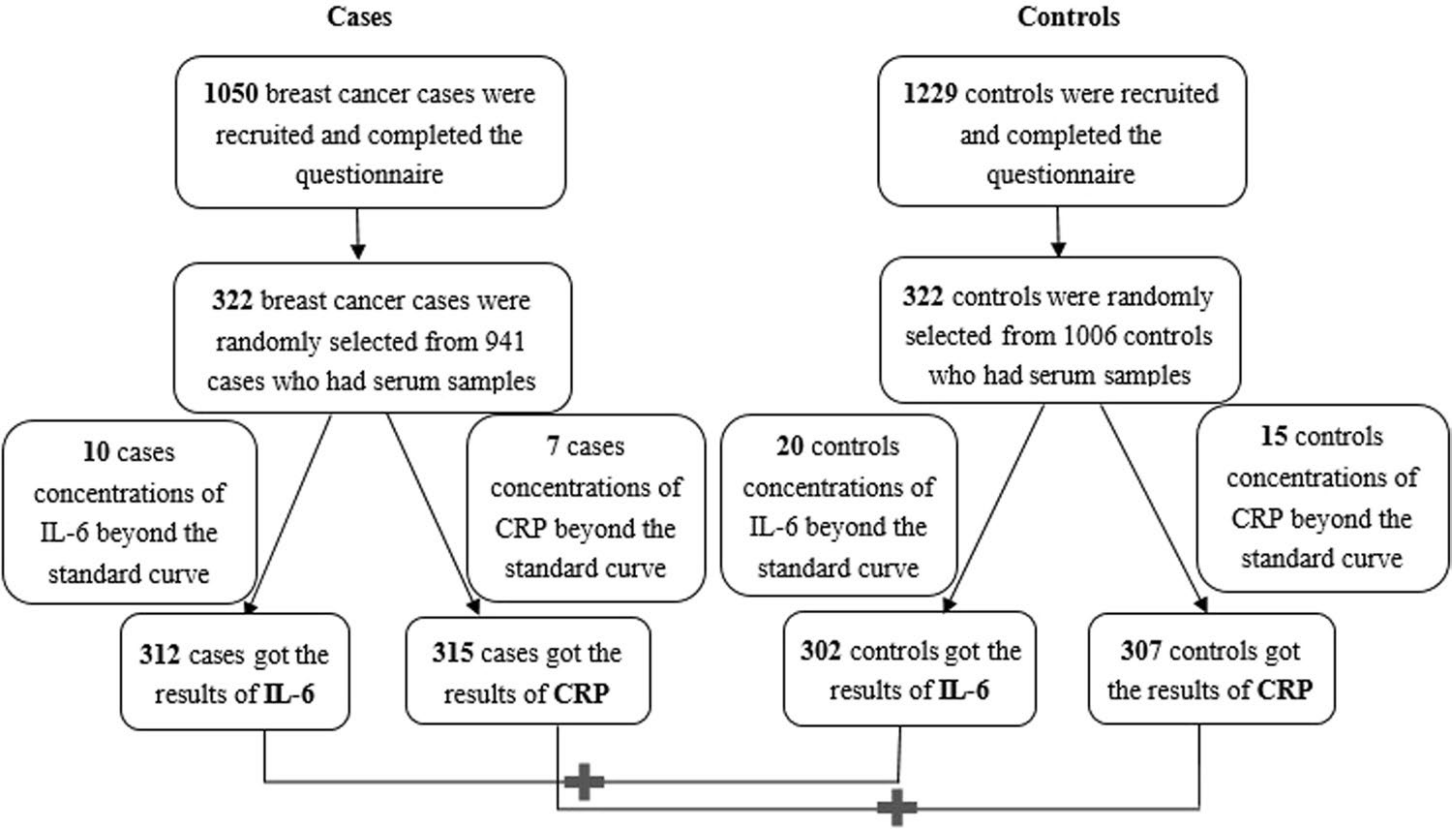
Flow chart of study participants (IL-6, Interleukin-6; CRP, C-reactive protein).

In this case–control study, 1050 case patients and 1229 control subjects completed a food frequency questionnaire (FFQ). Data on the diet and serum samples of 941 and 1006 of these cases and controls were available, respectively, and 322 cases and 322 controls were randomly selected from them. The serum samples of these selected participants were used to measure serum IL-6 and CRP levels. The IL-6 levels of 312 cases (96.9%) and 302 controls (93.79%) and CRP levels of 315 cases (97.83%) and 307 controls (95.34%) were within the standard curve of the assay, whereas those of the remaining cases and controls were beyond the standard curve (Fig. 1**)**.

### Comparison of baseline characteristics between cases and controls

As shown in Table 1, compared with controls, cases reached menarche at an earlier age and had higher body mass index (BMI). They were also more likely to have a lower educational level, history of passive smoking, history of first-degree relative with cancer, history of benign breast disease and more children, and were less likely to be physically active. Moreover, compared with controls recruited in the experiments, a higher number of cases had a lower income level, history of passive smoking, first-degree relative with cancer and benign breast disease. All of these variables were regarded as potential confounders and were adjusted for in the subsequent multivariable logistic analyses. Compared with controls, cases tended to have a higher intake of fat and a lower intake of magnesium, calcium, phosphorus and dietary fibre. However, no significant difference was found in the intake of energy and carbohydrate between them.

**Table 1:** Characteristics data of breast cancer cases and controls.

Variables	Samples recruited for questionnaire			Samples recruited for experiments		
	Cases (n = 1050)	Controls (n = 1229)	*P* value	Cases (n = 322)	Controls (n = 322)	*P* value
Age, years	47.79 ± 9.42	47.88 ± 9.48	0.817	48.3 ± 9.5	47.8 ± 9.8	0.513
Age at menarche, years	14.41 ± 1.90	14.75 ± 1.86	<0.001	14.8 ± 1.8	14.7 ± 1.7	0.742
Age at first live birth^a^, years	25.57 ± 3.79	25.46 ± 3.55	0.491	25.7 ± 3.7	25.5 ± 3.1	0.439
BMI, kg/m^2^	23.06 ± 3.26	22.60 ± 3.11	0.001	23.34 ± 3.35	22.54 ± 3.19	0.002
Marital status						0.869
Married	989 (94.2)	1155 (94.0)	0.831	302 (93.8)	303 (94.1)	
Unmarried/divorced/widowed	61 (5.8)	74 (6.0)		20 (6.2)	19 (5.9)	
Educational level			0.001			0.079
Primary school or below	273 (26.0)	336 (27.3)		81 (25.2)	81 (25.2)	
Junior high school	299 (28.4)	288 (23.4)		97 (30.1)	79 (24.5)	
Senior high school	255 (24.3)	286 (23.3)		82 (25.5)	72 (22.4)	
Secondary technical school	133 (12.7)	151 (12.3)		34 (10.6)	43 (13.4)	
College or above	90 (8.6)	168 (13.7)		28 (8.7)	47 (14.6)	
Occupation			0.377			0.534
Blue collar worker	284 (27.0)	323 (26.3)		75 (23.3)	84 (26.1)	
Administrator/other white collar worker	195 (18.6)	257 (20.9)		67 (20.8)	67 (22.4)	
Unemployed/other	571 (54.4)	649 (52.8)		180 (55.9)	166 (51.6)	
Income level (yuan/month)			0.949			0.032
≤2000	144 (13.7)	177 (14.4)		27 (8.4)	17 (5.3)	
2001~5000	289 (27.5)	328 (26.7)		95 (29.5)	76 (23.6)	
5001~8000	325 (31.0)	384 (31.2)		113 (35.1)	112 (34.8)	
≥8001	292 (27.8)	340 (27.7)		87 (27.0)	117 (36.3)	
Leisure-time physical activity			0.002			0.636
Never	446 (42.5)	446 (36.3)		135 (41.9)	124 (38.5)	
Seldom (1 time/week)	66 (6.3)	62 (5.0)		15 (4.7)	14 (4.3)	
Often (≥1 time/week)	538 (51.2)	721 (58.7)		172 (53.4)	184 (57.1)	
Breastfeeding history^b^	885 (84.3)	1070 (87.1)	0.325	264 (82.0)	274 (86.1)	0.289
Regular drinker	87 (8.3)	80 (6.5)	0.105	23 (7.1)	20 (6.2)	0.636
Regular smoker	15 (1.4)	13 (1.1)	0.536	2 (0.6)	2 (0.6)	0.367
Passive smoking	639 (60.9)	620 (50.4)	<0.001	198 (61.5)	153 (47.5)	<0.001
First-degree relative with cancer	179 (17.0)	91 (7.4)	<0.001	70 (21.7)	42 (13.0)	0.004
History of benign breast disease	387 (36.9)	267 (21.7)	<0.001	117 (36.3)	88 (27.3)	0.014
Menopausal status			0.551			0.514
Premenopausal	668 (63.6)	767 (62.4)		198 (61.5)	206 (64.0)	
Postmenopausal	382 (36.4)	462 (37.6)		124 (38.5)	116 (36.0)	
Parity			0.001			0.635
0	46 (4.4)	36 (2.9)		17 (5.3)	12 (3.7)	
1~2	762 (72.6)	830 (67.5)		236 (73.3)	239 (74.2)	
≥3	242 (23.0)	363 (29.5)		69 (21.4)	71 (22.0)	
Ever used an oral contraceptive	88 (8.4)	73 (5.9)	0.551	22 (6.8)	21 (6.5)	0.875
Hormone replacement therapy use	46 (4.4)	41 (3.3)	0.194	5 (1.6)	8 (2.5)	0.401
Magnesium, mg/d	211.9 (169.0,266.7)	227.2 (181.6,283)	<0.001	186.3 (149.8,234.4)	217.7 (180.7,268.0)	<0.001
Calcium, mg/d	364.1 (281.1,471.9)	413.3 (321.2,547.9)	<0.001	336.3 (253.5,438.6)	398.8 (324.0,522.0)	<0.001
Phosphorus, mg/d	827.8 (699.6,986.5)	855.9 (725.2,1019.6)	0.001	797.1 (640.3,937.9)	873.2 (737.2,1024.3)	<0.001
Dietary fiber, g/d	8.3 (6.7,10.3)	9.1 (7.4,11.3)	<0.001	8.2 (6.5,9.9)	8.9 (7.2,10,7)	<0.001
Energy, kcal/d	1373 (1178,1638)	1367 (1178,1637)	0.706	1340 (1140,1635)	1359 (1194,1618)	0.593
Carbohydrate, g/d	215.7 (184.2,258.2)	216.2 (188.4,265.3)	0.276	214.4 (184.2,254.5)	211.4 (190.2,262.7)	0.840
Protein, g/d	61.2 (49.8,72.6)	62.2 (51.3,74.7)	0.077	57.0 (46.6,66.9)	64.2 (53.1,75.9)	<0.001
Fat, g/d	55.3 (42.4,71.3)	52.8 (42.1,69.1)	0.040	60.3 (47.7,76.2)	51.2 (40.0,67.3)	<0.001
IL-6, pg/mL	/	/	/	1.8 (1.1,3.3)	1.6 (1.0,2.8)	0.034
CRP, ng/mL	/	/	/	2476.0 (912.0,5900.0)	2032.0 (768.0,5204.0)	0028
Sex hormone status^c^						
ER+ & PR+	447 (42.6)	/	/	195 (60.6)	/	/
ER− & PR−	210 (20.0)	/	/	80 (24.8)	/	/

Abbreviation: IL-6, interleukin-6; CRP, C-reactive protein; ER, estrogen receptor; PR, progesterone receptor. Age, age at menarche, age at first live birth and BMI were shown as mean ± standard deviations and *t*-test was used to test the differences between the case and control subjects. Magnesium, calcium, phosphorus, dietary fiber, energy, carbohydrate, protein, fat, IL-6 and CRP were shown as median (interquartile range) and Wilcoxon rank-sum test was used for the comparison between cases and controls. Categorical variables were shown as number (percentage) and Chi-square test was used to test the differences.

^a^Among women who had a live birth.

^b^Among breast-feeding women.

^c^Among cases.

### Association between dietary magnesium, IL-6, CRP and overall breast cancer risk

**Figure 2:**
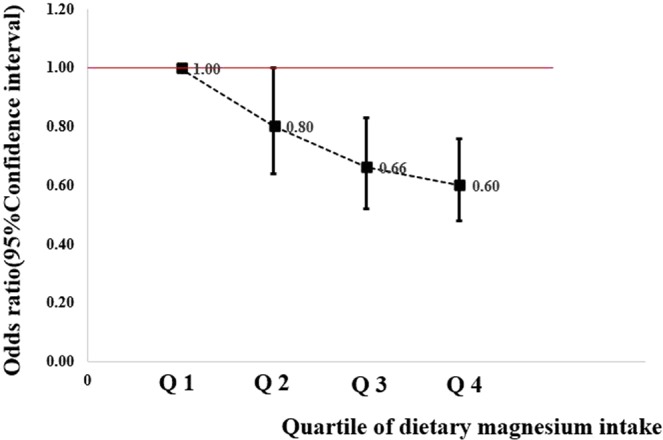
Dose–response relationship between magnesium intake and breast cancer risk in Chinese women.

The odds ratios (ORs) and 95% confidence interval (CIs) of dietary magnesium intake, IL-6 level, CRP level and overall breast cancer risk are shown in Table 2. A higher dietary magnesium intake was associated with a lower breast cancer risk (crude OR = 0.75, 95% CI = 0.62, 0.92). This association remained significant after adjusting for basic characteristics (OR = 0.78; 95% CI = 0.63, 0.95) in Model 2 and after further adjusting for dietary factors (OR = 0.80; 95% CI = 0.65, 0.99) in Model 3. As shown in Fig. 2, there was a trend of reduced risk of breast cancer associated with increasing magnesium intake (*P*-trend < 0.001). A marginal positive association was observed between the IL-6 level and breast cancer risk when IL-6 levels were categorised into two groups based on the 1.5-pg/mL cut-off value (crude OR = 1.38; 95% CI = 1.00, 1.90). However, the association became non-significant after adjusting for potential confounders. Compared with participants with CRP values ≤3000 ng/mL (the lower group), those with CRP values >3000 ng/mL (the higher group) had a 1.43 times higher breast cancer risk (95% CI = 1.04–1.97). The association in the adjusted model remained significant with an OR of 1.43 and a 95% CI of 1.02–2.01.

**Table 2:** Odds ratios (ORs) and 95% confidence intervals (95%CI) of dietary magnesium, interleukin-6, C-reactive protein and overall breast cancer risk in a Chinese case-control study, 2011–2016.

	Cases/controls (n)	Model 1^a^	Model 2^b^	Model 3^c^
**Magnesium**				
<280 mg/d	832/912	1.00	1.00	1.00
≥280 mg/d	218/317	0.75 (0.62,0.92)	0.78 (0.63,0.95)	0.80 (0.65,0.99)
*P* value		0.005	0.016	0.047
**IL-6**				
≤1.5 pg/mL	134/146	1.00	1.00	
>1.5 pg/mL	178/156	1.38 (1.00,1.90)	1.33 (0.95,1.87)	
*P* value		0.051	0.076	
**CRP**				
≤3000 ng/mL	174/196	1.00	1.00	
>3000 ng/mL	141/111	1.43 (1.04,1.97)	1.43 (1.02,2.01)	
*P* value		0.029	0.037	

Abbreviation: IL-6, interleukin-6; CRP, C-reactive protein.

^a^Values were showed as crude OR and 95%CI; IL-6 values ≤1.5 pg/mL as reference group; CRP values ≤3000 ng/mL as reference group; Magnesium values <280 mg/d as reference group.

^b^ORs of magnesium and breast cancer risk were adjusted for age at menarche, BMI, educational level, passive smoking, physical activity, parity, first-degree relative with cancer and history of benign breast disease. ORs of IL-6, CRP and breast cancer risk were adjusted for passive smoking, body mass index, first-degree relative with cancer, history of benign breast disease and income level.

^c^Adjusted for confounders from model 2 plus intakes of energy, fat, calcium, phosphorus and dietary fiber.

### Stratified analyses

The results of the stratified analyses according to menopausal status, BMI and sex hormone status are shown in Table 3. A negative association was found between dietary magnesium intake and breast cancer risk among premenopausal women in Model 3 (adjusted OR = 0.75; 95% CI = 0.57, 0.98), but not among postmenopausal women. Stratified analysis by sex hormone status showed that dietary magnesium intake had a significant negative association with oestrogen receptor (ER)-positive (ER+) and progesterone receptor (PR)-positive (PR+) breast cancer risk in Model 3 (adjusted OR = 0.71; 95% CI = 0.53, 0.95). After adjusting for potential confounders, stratified analysis by BMI showed that a higher dietary magnesium intake was associated with a lower breast cancer risk among overweight or obese women (Model 3: adjusted OR = 0.69, 95% CI = 0.48, 0.99), but not among women with normal weight. Irrespective of the amount of calcium intake, no association was found between dietary magnesium intake and breast cancer risk after stratification.

**Table 3:** Odds ratios (ORs) and 95% confidence intervals (95%CI) of dietary magnesium and breast cancer risk in stratified analyses.

Variables	Magnesium		*P* interaction^d^
	<280 mg/d	≥280 mg/d	
Menopausal status			0.016
**Premenopausal**			
No.Cases/controls	527/555	141/212	
Model 1^a^	1	0.70 (0.55,0.89)	
Model 2^b^	1	0.73 (0.56,0.94)	
Model 3^c^	1	0.75 (0.57,0.98)	
**Postmenopausal**			
No.Cases/controls	305/357	77/105	
Model 1^a^	1	0.86 (0.62,1.20)	
Model 2^b^	1	0.86 (0.61,1.22)	
Model 3^c^	1	0.90 (0.62,1.29)	
Sex hormone status			0.992
**ER+ & PR+**			
No.Cases/controls	367/912	80/317	
Model 1^a^	1	0.63 (0.48,0.82)	
Model 2^b^	1	0.69 (0.52,0.91)	
Model 3^c^	1	0.71 (0.53,0.95)	
**ER− & PR−**			
No.Cases/controls	168/912	42/317	
Model 1^a^	1	0.72 (0.50,1.03)	
Model 2^b^	1	0.80 (0.55,1.16)	
Model 3^c^	1	0.79 (0.53,1.17)	
Body mass index (BMI)			0.008
**Normal weight (BMI** ≥ **18**.**5&BMI** < **24)**			
No.Cases/controls	494/565	121/184	
Model 1^a^	1	0.75 (0.58,0.98)	
Model 2^b^	1	0.81 (0.62,1.07)	
Model 3^c^	1	0.83 (0.62,1.10)	
**Overweight or obese (BMI** ≥ **24)**			
No.Cases/controls	288/269	81/112	
Model 1^a^	1	0.68 (0.49,0.94)	
Model 2^b^	1	0.67 (0.48,0.95)	
Model 3^c^	1	0.69 (0.48,0.99)	
Calcium intake			0.001
**Low (**<**800** **mg/d)**			
No.Cases/controls	812/889	194/262	
Model 1^a^	1	0.81 (0.66,1.00)	
Model 2^b^	1	0.84 (0.67,1.04)	
Model 3^c^	1	0.83 (0.66,1.04)	
**High (**≥**800** **mg/d)**			
No.Cases/controls	24/55	20/23	
Model 1^a^	1	0.50 (0.23,1.08)	
Model 2^b^	1	0.54 (0.22,1.32)	
Model 3^c^	1	0.47 (0.18,1.22)	

^a^Values were showed as crude OR and 95%CI; Magnesium values <280 mg/d as reference group.

^b^ORs of magnesium and breast cancer risk were adjusted for age at menarche, BMI, educational level, passive smoking, physical activity, parity, first-degree relative with cancer and history of benign breast disease.

^c^Adjusted for confounders from model 2 plus intakes of energy, fat, calcium, phosphorus and dietary fiber.

^d^*P* value for interactive effect.

### Direct and indirect associations between dietary magnesium intake and breast cancer risk

**Figure 3:**
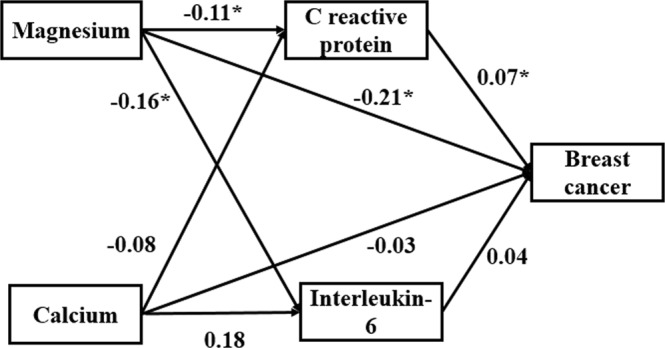
Path model of associations between magnesium and calcium intake, inflammatory marker levels (IL-6 and CRP) and breast cancer risk in Chinese women. Values are estimates of direct effects; *P value < 0.05.

Table 4 shows the total, direct and indirect effects of dietary magnesium and calcium intake on breast cancer risk. Figure 3 displays the path model and the estimates of direct effects. Posterior predictive *P* value in this model was 0.55, indicating that this model could appropriately fit the data. The total effects of magnesium and calcium intake on breast cancer risk were −0.22 (95% CI = −0.34, −0.11; *P* < 0.05) and −0.03 (95% CI = −0.22, 0.17; *P* > 0.05), respectively. Direct and indirect effects of dietary magnesium were statistically significant for breast cancer risk with estimates of −0.21 and −0.01, and the CRP level played a mediating role in the association between dietary magnesium intake and breast cancer risk.

**Table 4:** Total, direct and indirect effects of dietary magnesium and calcium intake in Path analysis of breast cancer.

	Magnisium		Calcium	
	Estimates	95% CI	Estimates	95% CI
Total effect	−0.22*	−0.34,−0.11	−0.03	−0.22,0.17
Direct effect	−0.21*	−0.32,−0.09	−0.03	−0.22,0.17
Indirect effect	−0.01*	−0.04,0	0	—

*P value < 0.05.

## Discussion

This study showed that dietary magnesium intake was inversely associated with breast cancer risk and that higher CRP level was a risk factor for breast cancer development. Path analysis revealed that the serum CRP level, but not the serum IL-6 level, mediated the association between dietary magnesium intake and breast cancer risk.

This study focused on the association between dietary magnesium intake and breast cancer risk because diet is an easy target for intervention. The results showed an inverse association between dietary magnesium intake and overall breast cancer risk. To date, few epidemiological studies have investigated the association between dietary magnesium intake and breast cancer risk. Consistent with our result, an Italian case–control study found that the serum magnesium level was significantly lower among breast cancer patients than among control subjects^2^. Magnesium deficiency has been found to be involved in both the risk and prognosis of cancers, including breast cancer^2,6,40,41^. Some studies have focused on the effect of dietary magnesium on the prognosis of breast cancer^9,10^. Their results suggest that higher dietary magnesium intake is inversely associated with mortality among breast cancer patients.

Several experimental studies have suggested that magnesium affects tumourigenesis through two mechanisms, namely inflammation and free radicals-induced oxidative stress, both of which may cause DNA damage, subsequently leading to tumour onset^41^. In particular, dietary magnesium has been reported to play a crucial role in the regulation of systematic chronic low-grade inflammation, especially the circulating CRP level^14–18^. A meta-analysis including seven cross-sectional studies suggested an inverse association between dietary magnesium intake and serum CRP levels^14^. Another meta-analysis of 11 randomised controlled trials indicated that magnesium supplementation reduces circulating CRP levels in individuals with inflammation (CRP levels >3000 ng/mL)^15^. These findings indicate that magnesium intake may play a protective role in the management of inflammation, which may be one of the mechanisms of tumourigenesis. Therefore, the present study also explored how dietary magnesium affects breast cancer risk through inflammation. This was the first study to explore dietary magnesium together with inflammatory markers as the risk factor for breast cancer. The results showed that dietary magnesium intake affected breast cancer risk both directly and indirectly by modifying the CRP level. This result supports the hypothesis proposed in previous experimental studies that the potential beneficial effect of dietary magnesium intake on breast cancer prevention may, at least in part, be explained by the inhibition of inflammation^41^.

The signalling pathways linked to both STAT3 and NF-κB have been suggested to play important roles in the communication between inflammatory cells and cancer cells^42,43^. In particular, IL-6 has been proven to activate NF-κB and STAT3 pathways to facilitate a tumour micro-environment^36,42,43^. However, there is little evidence from observational studies investigating the effect of IL-6 level on breast cancer risk^37–39^. Consistent with our result, a combined analysis of two prospective studies revealed no significant relation between the IL-6 level and overall breast cancer risk^39^. In contrast with our result, a Korean case–control study found that the IL-6 level was significantly higher in breast cancer patients than in control subjects^37^. Given these contradictory results, more epidemiological studies with larger sample sizes should be conducted to explore the effect of IL-6 level on breast cancer risk.

Fundamental experiments have revealed that the moderately high levels of pro-inflammatory markers in chronic inflammatory status are responsible for the formation of an inflammatory micro-environment, which affects the process of epithelial–mesenchymal transition^44,45^. CRP is a classic inflammatory marker that is highly sensitive to inflammatory response^24–26^. Several studies have attempted to investigate the association between the circulating CRP level and breast cancer risk, most of which have reported consistent results^30,33,35,38,39,46–52^; however, few such studies have been conducted on Chinese populations. To date, four meta-analyses have summarised previous studies^30,39,49,51^, three^30,49,51^ of which consistently supported a positive association between the CRP level and breast cancer risk as observed in our study. The other meta-analysis including 12 prospective studies suggested a marginal association between the CRP level and breast cancer risk^39^. A cohort study comprising 17 402 Chinese females showed that women with high CRP levels (>3000 ng/mL) at baseline had a significantly higher breast cancer risk (adjusted RR = 1.80; 95% CI = 1.03, 3.15) than women with low CRP levels (<1000 ng/mL)^52^. Thus, the results of the present study support the hypothesis that high CRP levels (>3000 ng/mL) are a risk factor for breast cancer among Chinese women.

The present study found an inverse relationship between dietary magnesium intake and breast cancer risk among overweight or obese women but not among women with normal weight. It is well-known that obesity is highly correlated with inflammatory response and increased free radical levels^53–56^. One plausible reason is that women with more adipose tissue are more sensitive to the effect of inflammation; thus, even if dietary magnesium has only a small anti-inflammatory effect, it is likely to affect breast cancer risk in overweight or obese women.

Stratified analyses by menopausal status revealed a negative relationship between dietary magnesium intake and breast cancer risk among premenopausal women. Oestrogen is derived mainly from the ovaries in premenopausal women and from aromatase in postmenopausal women; both of these sources have been found to be regulated by pro-inflammatory cytokines in distinct ways^57,58^. Differences in the oestrogen biosynthesis mechanisms may account for the different effects of inflammatory marker levels on breast cancer risk between pre- and postmenopausal women^57,59–61^. Dietary calcium intake has been suggested to have an interactive effect with magnesium on breast cancer risk. The protective effect of magnesium on breast cancer was stronger among women with adequate calcium intake than among those with calcium deficiency, although the association was non-significant partially due to the small sample size. Magnesium and calcium belong to the same family in the periodic table and share similar metabolic pathways^62^. A previous study on postmenopausal women suggested that compared with an adequate magnesium intake, dietary magnesium deficiency increases calcium retention^63^. The findings on the association between calcium intake and breast cancer risk remain controversial^64^.

To the best of our knowledge, this was the first study to explore the indirect effect of dietary magnesium on breast cancer via its influence on inflammatory marker levels and to assess the association between dietary magnesium intake and breast cancer risk among Chinese women. Despite its strengths, some limitations should be acknowledged. First, the sample size used for measuring inflammatory marker levels was relatively small, which may have affected the power of the test. Second, magnesium from hard water and supplements, which are also regarded as important sources of magnesium conducive to human body^17,65,66^, was not included in the calculation of dietary magnesium values. However, all study participants were natives of Guangdong province or had lived in Guangdong for at least 5 years, indicating that they shared similar water sources. In addition, we collected information about supplements and noted that few patients took magnesium supplements. Thus, magnesium from hard water and supplements may not have significantly affected the result. Third, selection bias and recall bias are inevitable in hospital-based case–control studies. To minimise selection bias, all control subjects were carefully recruited to exclude any diagnosis potentially related to breast cancer or dietary changes. In addition, the relatively high response rate also helped to reduce selection bias. To reduce recall bias, cases were interviewed immediately after breast cancer diagnosis. Moreover, food photographs were used to assist participants with the quantification of dietary intake. Fourth, it should be noted that it was impossible to include all mediators in the path model in which the estimates of direct and indirect effects might be affected. Moreover, the path model was based on the assumption that inflammatory response plays a mediating role, which should occur before the onset of breast cancer. However, this model could not completely avoid inverse causality due to the case–control design of the study. Thus, further prospective studies should be conducted to explore the mediating role of chronic inflammation in the association between dietary magnesium intake and breast cancer risk.

In conclusion, this study indicated that a higher dietary magnesium intake was associated with a lower breast cancer risk both directly and, in part, indirectly via reduction in the CRP level. The results also provided evidence of a positive association between the CRP level and breast cancer risk among Chinese women.

## Materials and Methods

### Study population

This hospital-based case–control study was conducted from September 2011 to July 2016, the details of which have been described elsewhere^67^. Study cases included patients admitted to the surgical units of the First Affiliated Hospital of Sun Yat-sen University and Guangdong Women and Children Hospital between September 2011 and July 2016 and were recruited based on their fulfilment of the inclusion and exclusion criteria. Patients were included if they were female, 25–70 years old, native of Guangdong province or had lived in Guangdong for at least 5 years and had been diagnosed with incident, primary, histologically confirmed breast cancer no more than 3 months before the study. Patients with a prior history of any cancer or who did not understand or speak Mandarin/Cantonese were excluded. Control subjects without breast cancer were frequency matched by age (5-year interval) to the case patients and were simultaneously recruited from the First Affiliated Hospital of Sun Yat-sen University. The remaining inclusion criteria for control subjects were similar to those for case patients. Control subjects were excluded if they were diagnosed with inflammatory disease, including chronic nasosinusitis, chronic otitis media, chronic tonsillitis or maxillary sinusitis.

This study followed the tenets of the Declaration of Helsinki, and all procedures involving humans were approved by the ethical committee of the School of Public Health, Sun Yat-sen University. All participants provided written informed consent for participation in the study before the interview.

### Data collection

During hospitalisation, all participants were interviewed in person by trained interviewers using a structured questionnaire that included questions on demographic information, lifestyle factors (e.g. regular smoking, passive smoking, regular drinking and leisure-time physical activity), self-reported weight and height, menopausal status, diseases, reproductive history and family history of cancer. Regular smoking was defined as ever smoking at least one cigarette per day for more than six consecutive months. Passive smoking was defined as exposure to second-hand tobacco smoke for at least 15 min per day during the previous year. Regular drinking was defined as drinking alcohol at least once per week during the previous year. Leisure-time physical activity was classified into never, seldom (1 time/week) and often (≥1 time/week). BMI was calculated by dividing weight (kg) by height squared (m^2^). Postmenopausal status was defined as at least 1 year since the last menstrual cycle. Relevant medical information, medical diagnosis, histological findings and ER and PR statuses were obtained from the hospital medical records.

A validated 81-item FFQ^68^ was used to collect the previous year’s dietary information before diagnosis for the cases or before the time of interview for controls. Magnesium, calcium, energy, macro-nutrients, phosphorus, and fibre intake per day was calculated from FFQ based on the frequency of food consumption, food items and serving sizes. Values of nutrients in foods were obtained from the 2002 Chinese Food Composition Table^69^.

### Laboratory measurement

Fasting venous blood samples of cases were collected on the second day of hospitalisation prior to any drug treatment or examination. The samples were centrifuged at 3000 rpm for 10 min at 4 °C, and the supernatants were aliquoted into eight parts of 200 μL. All serum samples were stored at −80 °C in an alarmed refrigerator for continuous monitoring until analysis.

High-sensitivity ELISA (Thermo Fisher Scientific, Inc., Carlsbad, CA) was used to measure IL-6 levels, and an ELISA kit (Thermo Fisher Scientific, Inc.) was used to measure CRP levels. Serum samples from both cases and controls were subjected to the same tests, run in the same batch of 96 samples (16 for standard curve, 40 for case samples and 40 for control samples), and assayed in a random order to reduce inter-assay variation and systematic bias. The mean intra- and inter-assay coefficients of variation were 4.9 and 6.0% for the IL-6 level and 5.0 and 5.0% for the CRP level, respectively.

### Statistical analysis

Based on Dietary Reference Intakes for Chinese residents^70^, magnesium levels were classified as low (<280 mg/day) or high (≥280 mg/day) based on EARs, and calcium levels were categorised as low (<800 mg/day) or high (≥800 mg/day) based on adequate intake values. According to the Centers for Disease Control and Prevention/American Heart Association criteria, CRP levels were classified as low (≤3000 ng/mL) or high (>3000 ng/mL); this classification was originally created for the risk assessment of cardiovascular disease and has subsequently been used to explore the effect of inflammation on the development of various diseases^14,15,27^. Similarly, IL-6 levels were also categorised as low (≤1.5 pg/mL) or high (>1.5 pg/mL). As no guideline is available for the categorisation of IL-6 levels, the average value among healthy populations obtained from data in the literature was chosen as the cut-off value^37,71^. T-test was used to determine the differences in age, age at menarche and age at first live birth between the cases and controls. Wilcoxon rank-sum test was used to evaluate the differences in magnesium, calcium, phosphorus, dietary fibre, energy, carbohydrate, protein, fat, IL-6 and CRP levels between the two groups. Chi-square test was used to compare the categorical variables between the groups. Multiple unconditional logistic regression analysis was used to estimate the OR and 95% CI of the associations between dietary magnesium intake, serum IL-6 level, serum CRP level and breast cancer risk. The groups of participants with the lowest levels were used as reference groups. The values of Model 1 were shown as crude OR and 95% CI. Model 2 was adjusted for age at menarche, BMI, educational level, passive smoking, physical activity, parity, first-degree relative with cancer and history of benign breast disease to investigate the association between magnesium intake and breast cancer risk. Model 3 was further adjusted for the confounders from Model 2 in addition to the intake of energy, fat, calcium, phosphorus and dietary fibre. The associations between IL-6 levels, CRP levels and breast cancer risk were examined after adjusting for passive smoking, BMI, first-degree relative with cancer, history of benign breast disease and income level. Confounders were selected by comparing baseline characteristics between the cases and controls and between the current study and previous studies that evaluated the risk factors for breast cancer. Dietary magnesium intake was also classified into quartiles to explore the dose–response relationship with breast cancer risk, and then, linear trend was evaluated by entering the median value of magnesium intake for each quartile in the multiple regression model. Stratified analyses by menopausal status (premenopausal and postmenopausal), sex hormone status [ER+, or ER-negative (ER−); PR+ or PR-negative (PR−)], BMI (normal weight: BMI ≥ 18.5 and <24 kg/m^2^; overweight or obese: BMI ≥ 28 kg/m^2^) and calcium intake (<800 mg/day and ≥800 mg/day) were also performed^72^. The interactive effect was calculated by including an interaction term in the multiple regression model.

Path analyses were performed to investigate whether the inflammatory factors IL-6 and CRP were potential mediators contributing to the associations between dietary magnesium intake, calcium intake and breast cancer risk in Chinese women. Maximum likelihood is the most popular method in path analysis that is based on the assumption of multivariate normality. In this study, Mardia’s coefficient of multivariate kurtosis was 13.66 and the critical ratio was 19.91. Both values less than 1.96 indicated significant non-normality; thus, Bayesian structural equation modelling was used to evaluate the overall presented path analysis, and estimations were conducted based on the Markov Chain Monte Carlo algorithm^73^. The model evaluation criterion was posterior predictive *P* value ranging from 0 to 1 with an acceptable quantity of 0.5 or close to it^74^.

All *P* values are two sided, and *P* values of <0.05 were considered as statistically significant. Statistical analyses were performed using SPSS 20.0 and AMOS 17.0.

## References

[1] Torre LA (2015). Global cancer statistics, 2012. CA Cancer J Clin.

[2] Sartori S (1992). Serum and erythrocyte magnesium concentrations in solid tumours: relationship with stage of malignancy. Magnes Res.

[3] Houston M (2011). The role of magnesium in hypertension and cardiovascular disease. J Clin Hypertens (Greenwich).

[4] Guerrero-Romero F, Jaquez-Chairez FO, Rodriguez-Moran M (2016). Magnesium in metabolic syndrome: a review based on randomized, double-blind clinical trials. Magnes Res.

[5] Ko HJ (2014). Dietary magnesium intake and risk of cancer: a meta-analysis of epidemiologic studies. Nutr Cancer.

[6] Blaszczyk U, Duda-Chodak A (2013). Magnesium: its role in nutrition and carcinogenesis. Rocz Panstw Zakl Hig.

[7] Nielsen FH (2010). Magnesium, inflammation, and obesity in chronic disease. Nutr Rev.

[8] Saris NE, Mervaala E, Karppanen H, Khawaja JA, Lewenstam A (2000). Magnesium. An update on physiological, clinical and analytical aspects. Clin Chim Acta.

[9] Yang CY (2000). Calcium and magnesium in drinking water and the risk of death from breast cancer. J Toxicol Environ Health A.

[10] Tao MH (2016). Associations of intakes of magnesium and calcium and survival among women with breast cancer: results from Western New York Exposures and Breast Cancer (WEB) Study. Am J Cancer Res.

[11] Nielsen FH (2016). Guidance for the determination of status indicators and dietary requirements for magnesium. Magnes Res.

[12] Rosanoff A, Weaver CM, Rude RK (2012). Suboptimal magnesium status in the United States: are the health consequences underestimated?. Nutr Rev.

[13] Glasdam SM, Glasdam S, Peters GH (2016). The Importance of Magnesium in the Human Body: A Systematic Literature Review. Adv Clin Chem.

[14] Dibaba DT, Xun P, He K (2014). Dietary magnesium intake is inversely associated with serum C-reactive protein levels: meta-analysis and systematic review. Eur J Clin Nutr.

[15] Simental-Mendia LE, Sahebkar A, Rodriguez-Moran M, Zambrano-Galvan G, Guerrero-Romero F (2017). Effect of Magnesium Supplementation on Plasma C-reactive Protein Concentrations: A Systematic Review and Meta-Analysis of Randomized Controlled Trials. Curr Pharm Des.

[16] Galland L (2010). Diet and inflammation. Nutr Clin Pract.

[17] Nielsen FH, Johnson LK, Zeng H (2010). Magnesium supplementation improves indicators of low magnesium status and inflammatory stress in adults older than 51 years with poor quality sleep. Magnes Res.

[18] Kim DJ (2010). Magnesium intake in relation to systemic inflammation, insulin resistance, and the incidence of diabetes. Diabetes Care.

[19] Balkwill F, Mantovani A (2001). Inflammation and cancer: back to Virchow?. Lancet.

[20] Grivennikov SI, Karin M (2011). Inflammatory cytokines in cancer: tumour necrosis factor and interleukin 6 take the stage. Ann Rheum Dis.

[21] Khan S, Shukla S, Sinha S, Meeran SM (2013). Role of adipokines and cytokines in obesity-associated breast cancer: therapeutic targets. Cytokine Growth Factor Rev.

[22] Dupuy AM (2003). Is C-reactive protein a marker of inflammation?. Nephrologie.

[23] Meguro S, Ishibashi M, Takei I (2012). [The significance of high sensitive C reactive protein as a risk factor for cardiovascular diseases]. Rinsho Byori.

[24] Gewurz H, Mold C, Siegel J, Fiedel B (1982). C-reactive protein and the acute phase response. Adv Intern Med.

[25] Ansar W, Ghosh S (2013). C-reactive protein and the biology of disease. Immunol Res.

[26] Ablij H, Meinders A (2002). C-reactive protein: history and revival. Eur J Intern Med.

[27] Pearson TA (2003). Markers of inflammation and cardiovascular disease: application to clinical and public health practice: A statement for healthcare professionals from the Centers for Disease Control and Prevention and the American Heart Association. Circulation.

[28] Doumatey AP, Zhou J, Adeyemo A, Rotimi C (2014). High sensitivity C-reactive protein (Hs-CRP) remains highly stable in long-term archived human serum. Clin Biochem.

[29] Zhang SM (2007). C-reactive protein and risk of breast cancer. J Natl Cancer Inst.

[30] Guo L (2015). C-reactive protein and risk of breast cancer: A systematic review and meta-analysis. Sci Rep.

[31] Zhou B (2014). C-reactive protein, interleukin-6 and the risk of colorectal cancer: a meta-analysis. Cancer Causes Control.

[32] Rocha P (2014). Prognostic impact of C-reactive protein in metastatic prostate cancer: a systematic review and meta-analysis. Oncol Res Treat.

[33] Guo YZ, Pan L, Du CJ, Ren DQ, Xie XM (2013). Association between C-reactive protein and risk of cancer: a meta-analysis of prospective cohort studies. Asian Pac J Cancer Prev.

[34] Xu M (2013). Serum C-reactive protein and risk of lung cancer: a case-control study. Med Oncol.

[35] Asegaonkar SB, Asegaonkar BN, Takalkar UV, Advani S, Thorat AP (2015). C-Reactive Protein and Breast Cancer: New Insights from Old Molecule. Int J Breast Cancer.

[36] Dethlefsen C, Hojfeldt G, Hojman P (2013). The role of intratumoral and systemic IL-6 in breast cancer. Breast Cancer Res Treat.

[37] Yeon JY (2011). Evaluation of dietary factors in relation to the biomarkers of oxidative stress and inflammation in breast cancer risk. Nutrition.

[38] Agnoli C (2017). Biomarkers of inflammation and breast cancer risk: a case-control study nested in the EPIC-Varese cohort. Sci Rep.

[39] Heikkila K (2009). Associations of circulating C-reactive protein and interleukin-6 with cancer risk: findings from two prospective cohorts and a meta-analysis. Cancer Causes Control.

[40] Castiglioni S, Maier JA (2011). Magnesium and cancer: a dangerous liason. Magnes Res.

[41] Wolf FI (2007). Magnesium and neoplasia: from carcinogenesis to tumor growth and progression or treatment. Arch Biochem Biophys.

[42] Fan Y, Mao R, Yang J (2013). NF-kappaB and STAT3 signaling pathways collaboratively link inflammation to cancer. Protein Cell.

[43] He G, Karin M (2011). NF-kappaB and STAT3 - key players in liver inflammation and cancer. Cell Res.

[44] Zhou C, Liu J, Tang Y, Liang X (2012). Inflammation linking EMT and cancer stem cells. Oral Oncol.

[45] Fisher DT, Appenheimer MM, Evans SS (2014). The two faces of IL-6 in the tumor microenvironment. Semin Immunol.

[46] Nelson SH (2017). The Association of the C-Reactive Protein Inflammatory Biomarker with Breast Cancer Incidence and Mortality in the Women’s Health Initiative. Cancer Epidemiol Biomarkers Prev.

[47] Ollberding NJ (2013). Prediagnostic leptin, adiponectin, C-reactive protein, and the risk of postmenopausal breast cancer. Cancer Prev Res (Phila).

[48] Frydenberg H (2016). Pre-diagnostic high-sensitive C-reactive protein and breast cancer risk, recurrence, and survival. Breast Cancer Res Treat.

[49] Wang J (2015). Plasma C-reactive protein and risk of breast cancer in two prospective studies and a meta-analysis. Cancer Epidemiol Biomarkers Prev.

[50] Dossus L (2014). C-reactive protein and postmenopausal breast cancer risk: results from the E3N cohort study. Cancer Causes Control.

[51] Chan DS, Bandera EV, Greenwood DC, Norat T (2015). Circulating C-Reactive Protein and Breast Cancer Risk-Systematic Literature Review and Meta-analysis of Prospective Cohort Studies. Cancer Epidemiol Biomarkers Prev.

[52] Wang G (2014). [Association between the level of high sensitivity C-reactive protein and risk of breast cancer among non-diabetic females: a prospective study in Kailuan group]. Zhonghua Zhong Liu Za Zhi.

[53] Cox AJ, West NP, Cripps AW (2015). Obesity, inflammation, and the gut microbiota. Lancet Diabetes Endocrinol.

[54] Mraz M, Haluzik M (2014). The role of adipose tissue immune cells in obesity and low-grade inflammation. J Endocrinol.

[55] Piva SJ (2013). Assessment of inflammatory and oxidative biomarkers in obesity and their associations with body mass index. Inflammation.

[56] Khoo NK (2012). Obesity-induced tissue free radical generation: an *in vivo* immuno-spin trapping study. Free Radic Biol Med.

[57] Samavat H, Kurzer MS (2015). Estrogen metabolism and breast cancer. Cancer Lett.

[58] Ziegler RG, Fuhrman BJ, Moore SC, Matthews CE (2015). Epidemiologic studies of estrogen metabolism and breast cancer. Steroids.

[59] Stachenfeld NS (2014). Hormonal changes during menopause and the impact on fluid regulation. Reprod Sci.

[60] Monteiro R, Teixeira D, Calhau C (2014). Estrogen signaling in metabolic inflammation. Mediators Inflamm.

[61] Brown KA, Simpson ER (2015). Estrogens, obesity, inflammation, and breast cancer-what is the link?. Semin Reprod Med.

[62] Iseri LT, French JH (1984). Magnesium: nature’s physiologic calcium blocker. Am Heart J.

[63] Nielsen FH, Milne DB, Gallagher S, Johnson L, Hoverson B (2007). Moderate magnesium deprivation results in calcium retention and altered potassium and phosphorus excretion by postmenopausal women. Magnes Res.

[64] Sahmoun AE, Singh BB (2010). Does a higher ratio of serum calcium to magnesium increase the risk for postmenopausal breast cancer?. Med Hypotheses.

[65] Makrides, M., Crosby, D. D., Bain, E. & Crowther, C. A. Magnesium supplementation in pregnancy. *Cochrane Database Syst Rev*, D937 (2014). 10.1002/14651858.CD000937.pub2 PMC6507506 24696187

[66] Tukiendorf A, Rybak Z (2004). New data on ecological analysis of possible relationship between magnesium in drinking water and liver cancer. Magnes Res.

[67] Zhang CX (2013). Choline and betaine intake is inversely associated with breast cancer risk: a two-stage case-control study in China. Cancer Sci.

[68] Zhang CX, Ho SC (2009). Validity and reproducibility of a food frequency Questionnaire among Chinese women in Guangdong province. Asia Pac J Clin Nutr.

[69] Yang, Y. X., Wang, G. Y. & Pan, X. C. *China Food Composition*. Peking University Medical Press 329 (Beijing, 2002).

[70] Chinese Nutrition Society. *Dietary Reference Intakes For Chinese Residents*. Chinese Light Manufacturing Press, (In Chinese) (Beijing, 2013).

[71] Cho HJ, Kivimaki M, Bower JE, Irwin MR (2013). Association of C-reactive protein and interleukin-6 with new-onset fatigue in the Whitehall II prospective cohort study. Psychol Med.

[72] Zhou BF (2002). Predictive values of body mass index and waist circumference for risk factors of certain related diseases in Chinese adults–study on optimal cut-off points of body mass index and waist circumference in Chinese adults. Biomed Environ Sci.

[73] Gajewski BJ (2006). Non-normal path analysis in the presence of measurement error and missing data: a Bayesian analysis of nursing homes’ structure and outcomes. Stat Med.

[74] Moghaddam HV, Asadi ZS, Akaberi A, Hashemian M (2013). Intimate partner violence in the eastern part of Iran: a path analysis of risk factors. Issues Ment Health Nurs.

